# Cultural Competence as a Predictive Factor of the Mental Resilience of Caregivers of Individuals with Disabilities

**DOI:** 10.1192/j.eurpsy.2026.11826

**Published:** 2026-07-31

**Authors:** V. C. Piletska, E. Kotrotsiou, A. Argyriadis, I. Andriopoulos, G. Charalampous

**Affiliations:** 1 Nursing Department, Frederick University, Nicosia, Cyprus; 2 Nursing Department, Hellenic Mediterranean University, Heraklion, Greece; 3 School of Health Sciences, Frederick University, Nicosia, Cyprus

## Abstract

**Introduction:**

Cultural competence and mental resilience are essential skills for caregivers of individuals with disabilities, particularly in multicultural settings and under conditions of long-term stress. While quantitative studies typically indicate high self-assessment in these domains, qualitative data may reveal more complex psychosocial dimensions.

**Objectives:**

To explore in depth the lived experiences of caregivers of individuals with disabilities regarding cultural competence and mental resilience, and to compare qualitative findings with corresponding quantitative assessments.

**Methods:**

Semi-structured interviews were conducted with 15 caregivers (primarily parents) of children or adults with disabilities. Thematic analysis was carried out using an interpretative approach. All participants had previously completed standardized questionnaires measuring cultural competence and mental resilience, which indicated high levels of self-reported scores.

**Results:**

The qualitative data revealed contradictions between the participants’ self-reported levels and their actual lived experiences. Many caregivers expressed difficulties in genuinely addressing cultural diversity and reported significant emotional strain, despite the high quantitative scores. Female caregivers showed signs of greater resilience, while active social participation was associated with more positive narratives and enhanced sense of meaning and self-efficacy.

**Conclusions:**

The discrepancy between quantitative and qualitative results highlights the need for a multidimensional assessment of caregiver wellbeing. Education in cultural competence and strengthening caregivers’ social inclusion may contribute to their psychological empowerment and help prevent emotional burnout.

**Disclosure of Interest:**

None Declared

